# Humane Slaughtering of Animals

**Published:** 1923-02

**Authors:** Nina Hamilton

**Affiliations:** Ferne, Donhead, Salisbury, Wilts; Ferne, Donhead, Salisbury, Wilts


### CORRESPONDENCE.

[Correspondence on all subjects is invited, but we cannot be responsible for the opinions expressed by our correspondents, who must give name and address as a guarantee of good faith, but not necessarily for publication. Correspondents are reminded that conciseness greatly facilitates early insertion.]

### Humane Slaughtering of Animals.

*To the Editor of* The Hospital and Health Review.

Sir,—There are few questions at present before the public which can compare in importance with that of slaughterhouse reform. There is the cruelty of killing animals by old-fashioned and inefficient methods, there is the insanitary condition of the slaughterhouse and consequent contamination of the meat, there is the wholesale poisoning of the meat itself by subjecting the animals to totally unnecessary suffering and distress. It is well known that a mother may poison her baby by the condition produced in her milk through anger or fright, and who can say how much the increase in cancer and other diseases is due to the large amount of pain-poisoned meat consumed in this country ?

We rightly pride ourselves on the advance we have made in animal protection, but in this matter this country is behind many others. In Switzerland, Sweden, and even Germany, a mechanically-operated stunning instrument is in general use, whereas with us in far the greater number of cases it is the pole-axe that is employed for the large animals and the knife for the smaller ones. The use of the pole-axe in killing large animals is objectionable because it often happens that the slaughterman strikes in the wrong place, and many blows are frequently delivered before the animal is brought down ; also the pole-axe has to be wrenched out of the head before the next blow is struck. The use of the knife without previous stunning is objectionable because consciousness of pain continues while the spinal cord and brain remain uninjured. But it is not only the actual act of killing which is cruel ; the manner in which cattle are driven to the point of death by kicks and blows and tail-twisting inflicts endless suffering. The horrible practice of killing one animal in front of another, of forcing them into the slaughterhouses streaming with blood and amid the reeking carcasses of the newly slain, of hanging pigs and calves up alive by their legs and then sticking them and bleeding them to death, cannot be defended by anyone who lays claim to a spark of humanity.

Recently I saw four bullocks killed by the Jewish method in the largest slaughterhouse in London, and the conditions were utterly revolting. There should be no private slaughterhouses, for privacy and secrecy always encourage carelessness and abuse. There is at present no law enforcing methods of humane killing as the standard for the whole country. There are no schools for slaughtermen ; boys and inexperienced men practice on the living animal, and generally speaking anyone may kill as he pleases and how he pleases. Complete reform involves the institution of public abattoirs instead of many private slaughterhouses, where the killing operations are continually under supervision of inspectors, where the building arrangements are such that the killing halls are large and spacious and the floors made of a material that can be washed clean. There can be no doubt that the meat of a bullock or any animal that has fought for its life amid the usual scenes of a private slaughterhouse is a danger to the health of those who eat it. Such meat is known in the trade as “ fevered.”

But first of all, and before far-reaching reforms can be carried, we need a short Act of Parliament making humane killing obligatory throughout the country. Such a measure has been drafted, and is being promoted by the Animal Defence Society, 35 Old Bond Street, W. The proposed legislation has already received considerable Parliamentary support, and I shall be glad to hear at the above address from anyone who is willing to support this crusade for mercy to helpless animals, which is also of vital importance to suffering humanity.

Nina Hamilton and Brandon.

Ferne, Donhead,

Salisbury, Wilts.

